# Supplementary material for: Patterns of Intron Gain and Loss in Fungi
Source: PLoS Biol. 2004 Nov 30;2(12):e422. doi: 10.1371/journal.pbio.0020422 (PMC532390; doi:10.1371/journal.pbio.0020422)
Supplement: Table S1 — Also available at http://genes.mit.edu/NielsenEtAl/. (4.3 MB ZIP). [file pbio.0020422.st001.zip › NielsenEtAl/html/371.html]

AN0734.1.NCU01634.1.MG06293.1.FG05491.1


```
 CLUSTAL W (1.82) Multiple Sequence Alignments - Introns Inserted


Sequence 1: MG06293.1	103 aa
Sequence 2: FG05491.1	103 aa
Sequence 3: NCU01634.1	107 aa
Sequence 4: AN0734.1	93 aa
Alignment Length: 107 aa
Number Identitical Residues: 90 aa
Alignment Score (without introns) 3362


MG06293.1 	MT----GR1GKGGK~GLGKGGAKRHRKILRDNIQGITKPAIRRLARRGGVKRISAM1IYE
NCU01634.1	MTRISSSG~GKGGK~GLGKGGAKRHRKILRDNIQGITKPAIRRLARRGGVKRISAM1IYE
FG05491.1 	MT----GR1GKGGK~GLGKGGAKRHRKILRDNIQGITKPAIRRLARRGGVKRISAM1IYE
AN0734.1  	MS------~---GR1-----GAKRHRKILRDNIQGITKPAIRRLARRGGVKRISAM~IYE
          	*:          *:      ************************************ ***

MG06293.1 	ETRGVLKSFLEGVIRDAVTYTEHAKRKTVTSLDVVYALKRQGR~TLYGFGG
NCU01634.1	ETRGVLKTFLEGVIRDAVTYTEHAKRKTVTSLDVVYALKRQGR~TLYGFGG
FG05491.1 	ETRGVLKTFLEGVIRDAVTYTEHAKRKTVTSLDVVYALKRQGR~TLYGFGG
AN0734.1  	ETRGVLKTFLEGVIRDAVTYTEHAKRKTVTSLDVVYALKRQGR1TLYGFGG
          	*******:*********************************** *******
```
